# Quantitative evaluation of coloration and functionalization efficiency for surface modification of regenerated cellulose fabrics

**DOI:** 10.1038/s41598-026-64814-z

**Published:** 2026-08-13

**Authors:** Rania Rashad Tawakol Gaafer, Heba A. Ameen, Heba Tolla El Sayed Abo El Naga, Manar Y. Abd El-Aziz, Basma M. Eid

**Affiliations:** 1 Spinning and Weaving Department, Faculty of Applied Arts, Damietta University, Damietta, Egypt; 2 Chemical Metrology Division, Materials Testing and Surface Chemical Analysis Lab, National Institute of Standards (NIS), Giza, Egypt; 3 Clothing and Knitting Industrial Research Department, Textile Research and Technology Institute, National Research Centre, 33 EL Buhouth St. (Former EL Tahrir St.), Dokki, Giza, Egypt; 4 Textile Research and Technology Institute, National Research Centre, 33 El Buhouth St., Dokki, Giza, Egypt

**Keywords:** Tencel and bamboo fabrics, Combined dyeing and finishing, Sustainable surface modification, Functional finishing, Natural dyes, Biotechnology, Chemistry, Environmental sciences, Materials science

## Abstract

Globally, the ever-growing demand for sustainable, functionalized cellulosic textile fabrics has increased, steering interest towards a new generation of eco-friendly regenerated cellulosic fibers. In this study, sustainable dyed and multi-functionalized Tencel and bamboo fabrics were developed via eco-friendly surface modification, followed by dyeing with natural dyes in the presence or absence of essential oils to fabricate multi-functionalized cellulosic dyeings in a single step. First, Tencel and Bamboo fabrics were modified using biopolymers, namely gelatin (10 g/L) /β-cyclodextrin (7 g/L) in the presence of citric acid/sodium hypophosphite as an environmentally benign crosslinking system via a pad–dry–cure technique. Subsequently, the pre-modified fabrics were dyed with turmeric dye or Natural Brown 6 dye with or without essential oils, namely clove, cinnamon, and tulsi (20 g/L). FT-IR analysis, scanning electron microscopy, and energy-dispersive X-ray spectroscopy, along with quantitative and qualitative evaluations, were performed. The results obtained revealed a significant improvement in antibacterial activity with bacterial reduction percentages exceeding 90%. In addition, excellent ultraviolet protection properties were achieved. The integration of natural dyes and essential oils within the biopolymers resulted in durable coloration, improved functionality, and enhanced performance properties while maintaining air permeability within a reasonable range. The developed approach represents an environmentally benign and effective strategy for producing added-value sustainable textiles with combined aesthetic and protective functionality.

## Introduction

Environmental concerns and growing demand for eco-friendly solutions are driving the textile sector toward sustainability^1^. The development of sustainable multifunctional textiles has become crucial, since the market for functional textiles is anticipated to grow from US$3.5 billion in 2025 to US$5.0 billion by 2032, with a compound annual growth rate (CAGR) of 5.2% between 2025 and 2032. This growth will be driven by rising demands for textile materials with enhanced performance, advanced textile finishing technologies, and consumer preferences for multifunctional, eco-friendly alternatives to traditional textiles^2^.

Therefore, the use of materials derived from cellulose has garnered a lot of attention in current trends in sustainable development^3^. Within this context, regenerated cellulose fibers have the potential to create highly efficient, environmentally friendly textile goods by combining the adaptability and safety of natural fibers with the comfort, renewability, and biodegradability of synthetic fibers^4^.

Bamboo has garnered significant attention recently as a green, environmentally friendly natural material^5^. Due to its lightweight, rapid growth, moisture resistance, adaptability to various climates, low density, and structural rigidity, bamboo is an exceptional natural material that offers major structural advantages^6^. Bamboo is a cheap and abundant natural resource that can be used to produce regenerated bamboo fiber. Bamboo fiber is derived from bamboo pulp, which is extracted by wet spinning from the bamboo stem and leaves. The process of hydrolysis-alkalization and multi-phase bleaching in the extraction of bamboo pulp is similar to that of viscose rayon fiber^7^. Moreover, lyocell fiber has emerged as the most successful type of regenerated cellulose fiber during the last decades^8^. In tandem with the viscose method, the Lyocell process was proposed. Courtaulds invented the method for generating staple fibers under the brand Tencel®, and today Lenzing in Austria produces similar fibers under the brand Lyocell®^9^. The term “Tencel” refers to the commercial name for Lyocell fibers, which are cellulose materials created by spinning wood pulp in a solvent solution containing N-Methylmorpholine-N-oxide (NMMO), avoiding the serious pollution problem associated with the viscose formation process^10^. Over 99% of the solvent is recycled within the process, making the fiber production environmentally acceptable^11^.

On the other hand, shifting towards sustainable colorants and eco-friendly finishing agents to replace synthetic dyes and harsh traditional chemicals has become one of the prominent research areas.^12,13^. In this context, a wide range of natural dyes, plant extracts, essential oils, and biopolymers,^14^ and other green bioactive agents have been explored to develop alternative dyeing, printing, and/or finishing methodologies that produce dyed/printed multi-functionalized fabrics while minimizing environmental impacts.

Many natural dyes have been utilized for the coloration and functionalization of textile fabrics. Such natural dyes have antibacterial properties, UV-protection activity, and other functional properties due to the presence of tannin, quinines, anthraquinones, ketone, and flavones in their composition^15^. Generally, natural dyes can be categorized into two classes: the first class includes curcumin and indigo dyes, and is called substantive dyes, while the second class is considered mordant natural dyes, where a proper mordant should be used during the dyeing process^1,15,16^. The mordant can be metallic, such as iron, copper, and aluminum, or bio-mordanting, for example, tannin, pomegranate peel, and natural polyphenol^17^. The mordanting process usually uses a single mordant or a combination of metallic mordant and bio-mordant. Studies investigated biopolymers, such as chitosan, as bio-mordants in the presence and absence of metal salts^18^.

On the other hand, the use of Essential oils (EOs) has attracted attention from researchers across multiple industries due to their natural containment of diversified functional properties^19^. The EOs are complex mixtures of volatile compounds that plants generate as secondary metabolites. Essential oils possess a unique composition of active compounds, aromatic activity, and a variety of biological actions^20^. They have been widely used in numerous industries, including cosmetics, food, packaging, medicine, and textiles^21^. In the textile finishing field, several EOs have been used as functional finishing agents to impart antimicrobial, UV-protective, aroma, and insect-repellent functionalities^21^. These essential oils include, but are not limited to, tulsi, clove, neem, cinnamon, and lavender^21–23^. However, the applications of EOs remain limited due to their hydrophobicity, volatility, and the instability of their chemical compounds. Additionally, it is worth mentioning that using natural dyes and essential oils in textile finishing is usually carried out individually^21,24^.

Green surface modifications of textile fabrics are performed to create new functionalities in textile materials, with ecological concerns in mind. It can be achieved by employing several techniques, ranging from simple processes such as the pad-dry-cure method to more innovative methodologies, including layer-by-layer deposition^25^, plasma activation^26,27^, biological approach^28^, chemical vapor deposition^29^, and others. Surface modification by coating textile materials with biomaterial films such as chitosan, starch, gelatin, cyclodextrin, etc., enabled the creation of new enhanced functionalized surfaces^30,31^.

Cyclodextrins (CDs) are cyclic oligosaccharides produced by enzymatic degradation of starch using the glucosyltransferase enzyme. CDs can form inclusion complexes with a variety of organic compounds such as fragrances, oils^32,33^, and dyes^34,35^. On the other hand, gelatin has emerged as an effective bio-mordant capable of modifying cellulose surface chemistry by introducing amino and carboxyl groups, which in turn can facilitate ionic interactions with anionic natural dye components. The utilization of these biopolymers for enhancing the efficiency of natural dyeing has been preliminarily investigated. β-CD was grafted onto cotton fabric using citric acid in the presence of sodium hypophosphite. The grafted cotton fabric was then dyed with natural coumarins. The results showed enhancement in UV protection property and crease recovery angle, along with a decrease in tensile strength^36^. Another study was carried out to investigate the effect of low-temperature plasma (LTP) treatment and grafting of beta-cyclodextrin (β-CD) on dyeing and fastness properties of wool fabrics using a natural dye extracted from shrimp shell, in the presence of several metallic mordants individually. The results obtained demonstrated that the β-CD-treated samples exhibited very good light and washing fastness properties^37^. β-cyclodextrin was grafted onto silk yarn using succinic acid and sodium hypophosphite, then post-dyed with several natural dyes including madder and turmeric dyes. The results revealed that the color strength of grafted samples was remarkably enhanced and exhibited high fastness properties. On the other hand, a study showed that the gelatin-tannic acid bio-mordanting system enhanced the color fastness of cotton fabric dyed with natural dyes^38^.

Therefore, this paper investigates the synergistic effect of gelatin/β-cyclodextrin as a bio-mordanting/host–guest inclusion system to enhance post-combined dyeing with natural dyes and finishing with essential oils of nominated regenerated cellulosic fabrics for developing sustainable multifunctional textile products. To achieve the goal, this research work is designed as follows: i) modification of Tencel and bamboo fabrics with gelatin and β-cyclodextrin in the presence of citric acid and sodium hypophosphite as a crosslinking system using pad-dry-cure to form a stable layer, ii) post-dyeing with natural dyes, namely turmeric dye and Natural brown 6, alone or in combination with certain essential oils. Characterization and full evaluation of the dyed/finished fabric samples were carried out.

## Materials and methods

### Materials

Plain-weave Tencel (99 gm/m^2^, fiber count No. 30/1 weft, 60/1 warp) and plain-weave bamboo viscose (97 gm/m^2^, fiber count No. 30/1 weft, 60/1 warp) were manufactured at CSA Textile, S.A.E., Egypt (a member of Ablioglu Holding Group, Turkey).

β-Cyclodextrin (99%) supplied by Acros Organics® with molecular weight 1135.01. Gelatin powder with molecular weight 40,000–50,000 supplied by Sigma-Aldrich, along with citric acid (CA), and Na-hypophosphite (SHP, NaH_2_PO_2_.H_2_O) were of laboratory reagent grade.

Kanga dye (Harda, Natural Brown 6) was obtained from ALPS Industries Ltd, Sahibabad, India. Turmeric dye powder and essential oils (namely cinnamon, clove, and basil) were obtained from the local market in Egypt. Natural dyes and essential oils were used throughout this study as received, without any further purification.

### Surface modification of cellulosic fabric using gelatin/cyclodextrin biopolymers

Samples of Tencel and bamboo viscose fabrics were padded twice (two dips and two nips), and squeezed with 80% wet pick-up in a solution containing functional finishing formulations that included 25 g/l citric acid as an eco-friendly polycarboxylic cross-linking agent and 12.5 g/l sodium hypophosphite as a catalyst. 7 g/l β-cyclodextrin, a natural cyclic-oligosaccharide, and 10 g/l gelatin, a biodegradable material with many functional side groups, were added to 100 ml of deionized water. The fabric samples were then cured for 5 min by microwave thermosfixation at 400 Watt.

### Post-concurrent dyeing and functional finishing

Portions of pre-modified regenerated cellulosic samples were simultaneously dyed and functional finished using a 50:1 liquor ratio and the exhaustion process, dyeing was carried out in an ultrasonic water bath with 60% amplitude, at a dyeing temperature 70 °C for 30 min, with a dyeing solution containing 10 g/l of turmeric powder or Natural dye 6 as natural dyes without and with 25 g/l different type of essential oil namely clove, cinnamon, or basil (the used oil was previously added to 1 g/l polysorbate/tween 80 as an emulsifier agent to enhance the solubility). The fabric samples were then cured for 5 min by microwave thermofixation at 300 Watt.

### Characterization

The untreated and surface-modified fabric samples using β-Cyclodextrin/gelatin biopolymers, followed by dyeing with natural dyes, were characterized using Fourier transform infrared spectroscopy. The analysis was performed using a Nicolet 380 spectrophotometer (Thermo Scientific). The spectra were acquired over a wavenumber range of 4000–500 cm^−1^.

The morphological surface of selected samples was studied using a scanning electron microscope (SEM) (Quanta SEM 250 FEG (Field Emission Gun) equipped with an energy–dispersive X-ray spectroscopy (EDX) with an accelerating voltage of 30 kV, FEI Co., Netherlands for the surface composition analysis. Scanning electron microscopy (SEM) and energy-dispersive X-ray spectroscopy (EDX) were employed to monitor changes in surface morphology and verify the elemental composition of the post-dyed/finished fabric samples, respectively.

### Analysis and testing

Nitrogen content (%N) of untreated, modified, and simultaneously dyed/functionalized fabric samples was measured using the Kjeldahl method .

Color strength (K/S) of the obtained dyeings was measured using a UV-Color-Eye 3100 spectrophotometer by employing the Kubelka–Munk equation . K/S = (1-R)^2^/2R (K: absorption coefficient, S: scattering coefficient, and R: reflectance of the dyed samples).

Color fastness to rubbing was evaluated according to ISO 105-X12 using an automatic crockmeter (Atlas), where dry and wet white cotton cloths were rubbed against the fabric surface. Color fastness to washing was assessed following ISO 105-C02, using a solution of 2 g/L nonionic surfactant at a material-to-liquor ratio of 1:50. The samples were treated for 40 min at 45 °C in a Rota machine, then rinsed, dried, and evaluated for color change.

The efficiency of imparted UV-resistance to regenerated cellulosic fabrics before and after dyeing and finishing was assessed through calculation of UV-protection factor (UPF) according to AS/NZS 4366:1996 using UV–vis spectrophotometer V-750, JASCO, Japan.

The antibacterial activity of simultaneously dyed and functional finished fabric samples against pathogenic *Staphylococcus aureus* (gram-positive, G + ve) and *Escherichia coli* (gram-negative, G-ve) bacteria was evaluated using qualitative and quantitative methods. A qualitative procedure was carried out according to the standard method AATCC 147–2011, while a quantitative assessment was conducted by calculating the percent reduction of bacterial colonies. R (%) = (B-A)/B × 100, according to the AATCC Test Method 100–1999. R is the reduction of tested bacterial colonies; A is the number of surviving bacteria of pre-modified and/or dyed and finished samples, and B is the number of existing colonies of untreated regenerated cellulosic samples.

The change in wrinkle resistance performance is evaluated by determining the Crease recovery angle (CRA). CRA was assessed for all the samples in the dry state, according to ISO 2313**-**1:2021 and calculated as follows [CRA (w + f)^o^], where w is the crease angle in the warp direction, and f is the crease angle in the weft direction.

The change in the soft-touch feel is evaluated by measuring the Surface roughness. Samples were measured before and after the treatment procedure using the Surface roughness tester model SA6260 supplied by SAMA Tool company, Italy.

The effect of treatment formulation on the air permeability (AP) of samples was evaluated using the Air Permeability Tester model M021A, from SDL-ATLAS, according to the test method ASTM D737-18:2023. The test was conducted under a test pressure of 200 Pa. The test sample area was 20 cm^2^.

Tensile properties of untreated and treated fabric samples, expressed as maximum load, were measured according to the standard method ASTM D5035, using an Instron tensile machine (Model 3345) with a force capacity of 5 kN.

Durability to washing was evaluated according to [61(2A)-1996: Colorfastness to Laundering, Home and Commercial: Accelerated (AATCC, 2002). Laundering conditions outlined in Test 2A: for fabrics that are expected to withstand repeated low-temperature machine washings] were followed.

All tests were performed in triplicate, and mean values and standard deviation were calculated.

## Results and discussion

### FTIR analysis

**Fig. 1:**
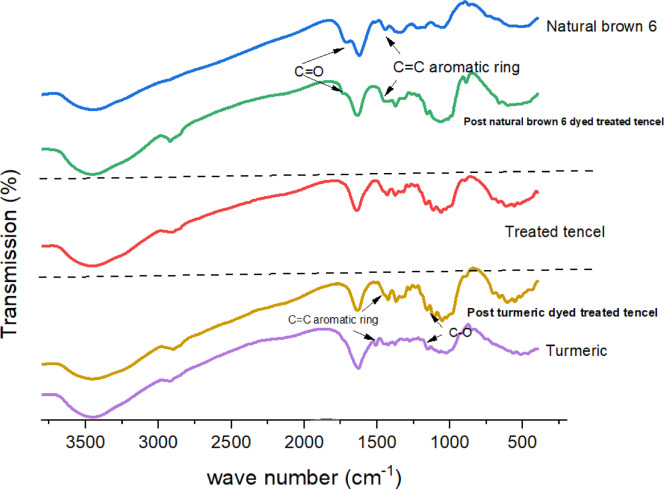
(**a**) FTIR spectrum of Blank Tencel fabric and treated Tencel fabric with gelatin and β-cyclodextrin (**b**) turmeric, post-turmeric dyed treated Tencel, Natural Brown 6, and Post Natural brown 6 treated Tencel.

The FT-IR spectrum of untreated Tencel fabric is presented in Fig. 1 and Table 1. The characteristic absorption bands of the cellulose glucose units are clearly observed in Fig. 1a. A broad band at approximately 3337 cm^−1^ is attributed to O–H stretching vibrations, while the band around **2**878 cm^−1^ corresponds to C–H stretching. The absorption at 1638 cm⁻^1^ is associated with adsorbed water molecules. The peak at 1464 cm^−1^ arises from symmetric bending vibrations of C–H and H–C–H groups. Additionally, the band at 1043 cm^−1^ is assigned to asymmetric in-plane C–O–C ring stretching, whereas the characteristic β-1,4-glycosidic (C1–O–C4) linkage appears at approximately 900 cm^−1^ due to asymmetric out-of-phase ring stretching^39^. The FT-IR spectra of Tencel fabric treated with gelatin and β-cyclodextrin using citric acid as a crosslinker. Figure 1a displays characteristic absorption bands corresponding to O–H, C–H, and C–C functional groups, confirming that the cellulose backbone of Tencel remains intact after the treatment process. The incorporation of gelatin and β-cyclodextrin introduces N–H vibrations and O–H at 3286 cm^−^1, which overlap with the O–H stretching region due to the close proximity of their vibrational wavenumbers^39^.

**Figure:**
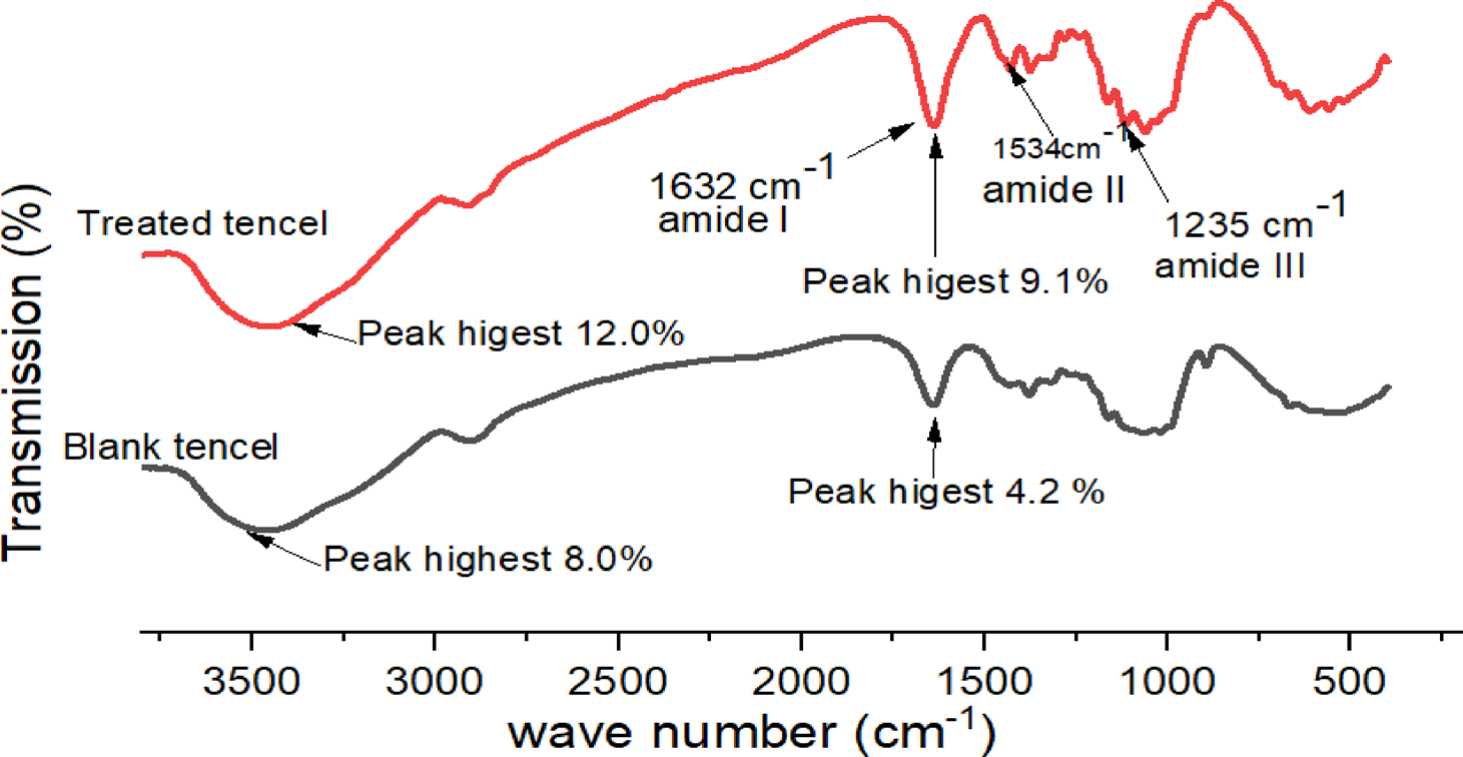

**Table 1:** Peak characterizations of post-dyed Tencel fabric with turmeric or Natural Brown 6.

Wave number (cm^-1^)	Functional group	Peak Highest (%)	References
Untreated Tencel fabric			
3337	O–H stretching vibration of hydroxyl groups of cellulose glucose units	8.0	^39^
2878	C–H stretching	2.3	
1638	O–H bending	4.2	
1464	H–C–H Symmetric bending vibration	1.1	
1043	C–O–C asymmetric ring stretching	1.0	
900	β-1,4-glycosidic linkage C1–O–C4 asymmetric	1.2	
Treated Tencel (gelatin/β-CD)			
3286	O–H / N–H stretching Overlapping O–H of cellulose and N–H vibrations of gelatin	12.0	^40–42^
1632	Amide I: C = O stretching vibration (protein backbone) overlapping with OH bending of cellulose and β-cyclodextrin	9.1	
1535	Amide II: N–H bending and C–N stretching	2.1	
1236	Amide III: C–N stretching and N–H bending	1.8	
Turmeric powder			
3328	O–H stretching Phenolic hydroxyl groups	7.2	^43^
2963	C–H stretching aliphatic C–H vibrations	2.1	
1635	C = O stretching Conjugated carbonyl group	5.8	
1510	C = C aromatic aromatic ring vibrations	3.4	
1226	C–O stretching aromatic ether vibrations	1.7	
Post-turmeric dyed treated Tencel fabric (New functional group on Tencel fabric)			
1509	C = C aromatic aromatic ring vibrations	**1.0**	
1225	C–O stretching Aromatic ether vibrations	**1.2**	
Natural Brown 6			
3587	O–H stretching Phenolic and carboxylic hydroxyl groups	5.1	^44^
1711	C = O stretching	3.8	
1642	aromatic C = C stretching vibrations	7.2	
1515	aromatic ring	2.4	
Post Natural Brown 6 Dyed treated Tencel fabric (New Functional group on Tencel fabric)			
1705	assigned to C = O stretching vibrations of carbonyl groups	2.1	
1523	aromatic ring	1.0	

Notably, the O–H stretching band shifts from 3337 cm^−1^ with a transmittance intensity of 8% in untreated Tencel to 3286 cm^−1^ with an increased intensity of 12% after treatment. The increase in intensity in Table 1,^40^ indicates the formation of stronger and more extensive intermolecular hydrogen bonding among cellulose, gelatin, and β-cyclodextrin. The enhanced intensity further suggests an increased abundance of hydroxyl groups, confirming the successful incorporation of β-cyclodextrin and gelatin onto the Tencel fabric surface.

Furthermore, the characteristic band at 1638 cm^−1^ with an intensity of 4.2% in untreated Tencel shifts to 1632 cm^−1^ with a significantly increased intensity of 9.1% after treatment Table 1. This change is attributed to the overlap between the cellulose-related vibration and the Amide I band (C = O stretching vibration) of gelatin. In addition, the appearance of new absorption bands at 1535 cm cm^−1^ and 1236 cm^−1^ corresponds to Amide II and Amide III vibrations, respectively, providing further evidence for the successful incorporation of gelatin into the treated Tencel fabrics.

**Fig. 2:**
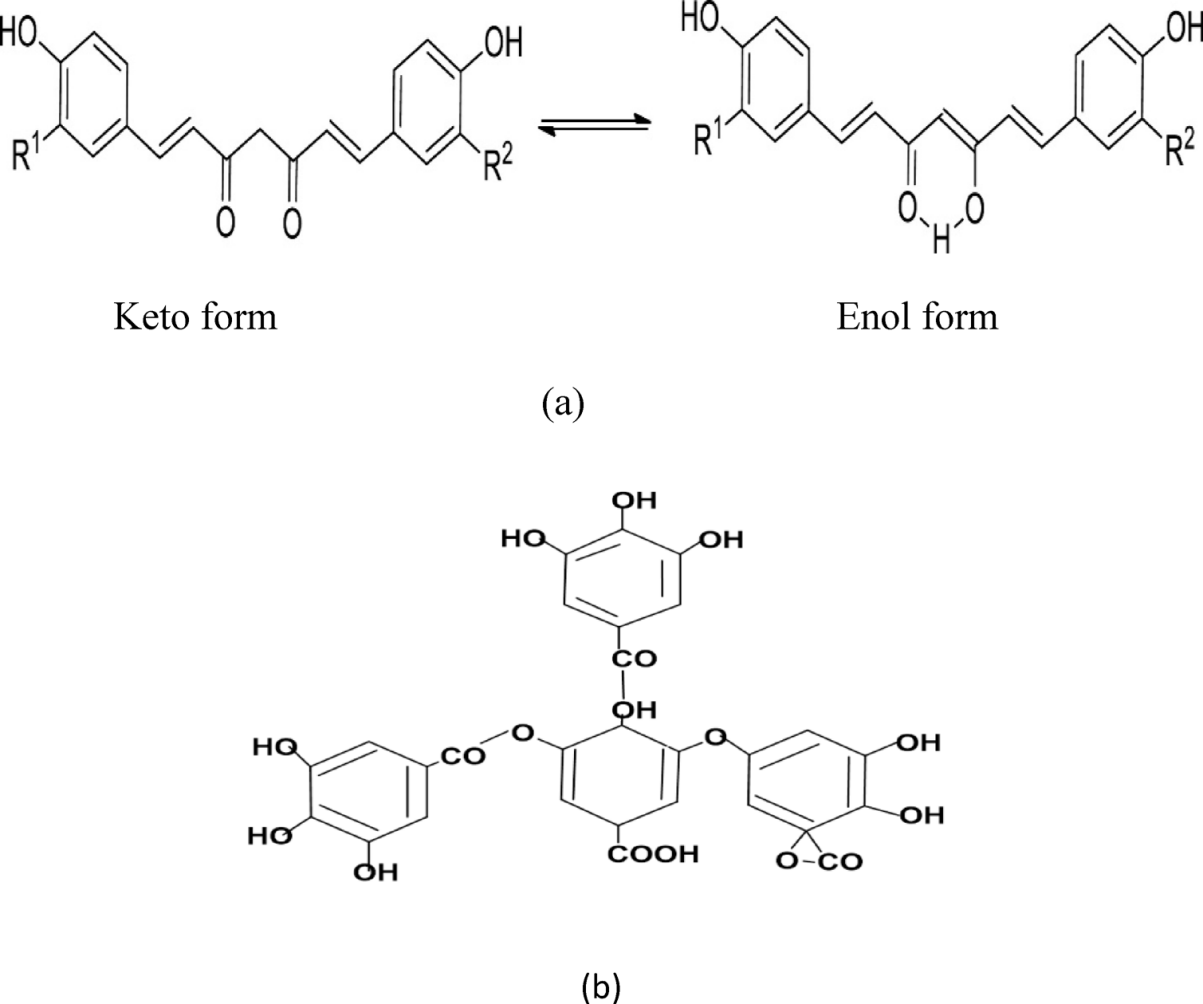
(**a**) Structure of Turmeric compound. (**b**) Structure of Natural Brown 6.

The FTIR spectrum Fig. 1b of turmeric, with structure as Fig. 2a shows characteristic bands at 3328 cm^−1^ (O–H stretching), 2963 cm^−1^ (C–H stretching), and 1635 cm cm^−1^ (conjugated C = O), along with aromatic ring vibrations at 1510 and 1226 cm^−1^. For the post-turmeric, Fig. 2a dyed Tencel fabric treated with gelatin and β-cyclodextrin, new absorption bands appeared at 1509 cm^−1^ and 1225 cm^−1^ for aromatic rings, which were absent in both untreated and treated Tencel fabric. The appearance of these bands confirms the successful binding and incorporation of turmeric onto the treated Tencel fabric via gelatin and β-cyclodextrin interactions, indicating the formation of hydrogen bonding interactions between turmeric and amid and hydroxyl groups of gelatin and cellulose.

On the other hand, Natural Brown 6 (Fig. 2b) is a natural dye extracted from the fruits of Terminalia chebula, in which chebulinic acid constitutes the major coloring component. The FT-IR spectrum of chebulinic acid (Terminalia chebula extract) exhibits a broad absorption band at 3587 cm^−1^, corresponding to O–H stretching vibrations of phenolic hydroxyl and carboxylic acid groups, indicative of strong hydrogen-bonding interactions. The absorption peak at 1711 cm^−1^ is attributed to C = O stretching vibrations of ester and carboxylic acid groups. Additionally, the band at 1642 cm^−1^ is associated with aromatic C = C stretching vibrations and conjugated carbonyl interactions, while the peaks appearing in the range of 1515 cm^−1^ correspond to aromatic ring skeletal vibrations.

The characteristic functional groups of Natural Brown 6 dye were clearly observed in the FT-IR spectrum of the dyed Tencel fabric after treatment. A noticeable shift in the O–H stretching band of the treated Tencel fabric toward higher wavenumbers indicates the formation of intermolecular hydrogen bonding between the treated fabric and the Brown 6 dye. Furthermore, the appearance of a new absorption band at approximately 1705 cm^−1^, assigned to C = O stretching vibrations of carbonyl groups, confirms the successful adsorption and fixation of the Brown 6 dye onto the Tencel substrate.

### Surface morphology study

**Fig. 3:**
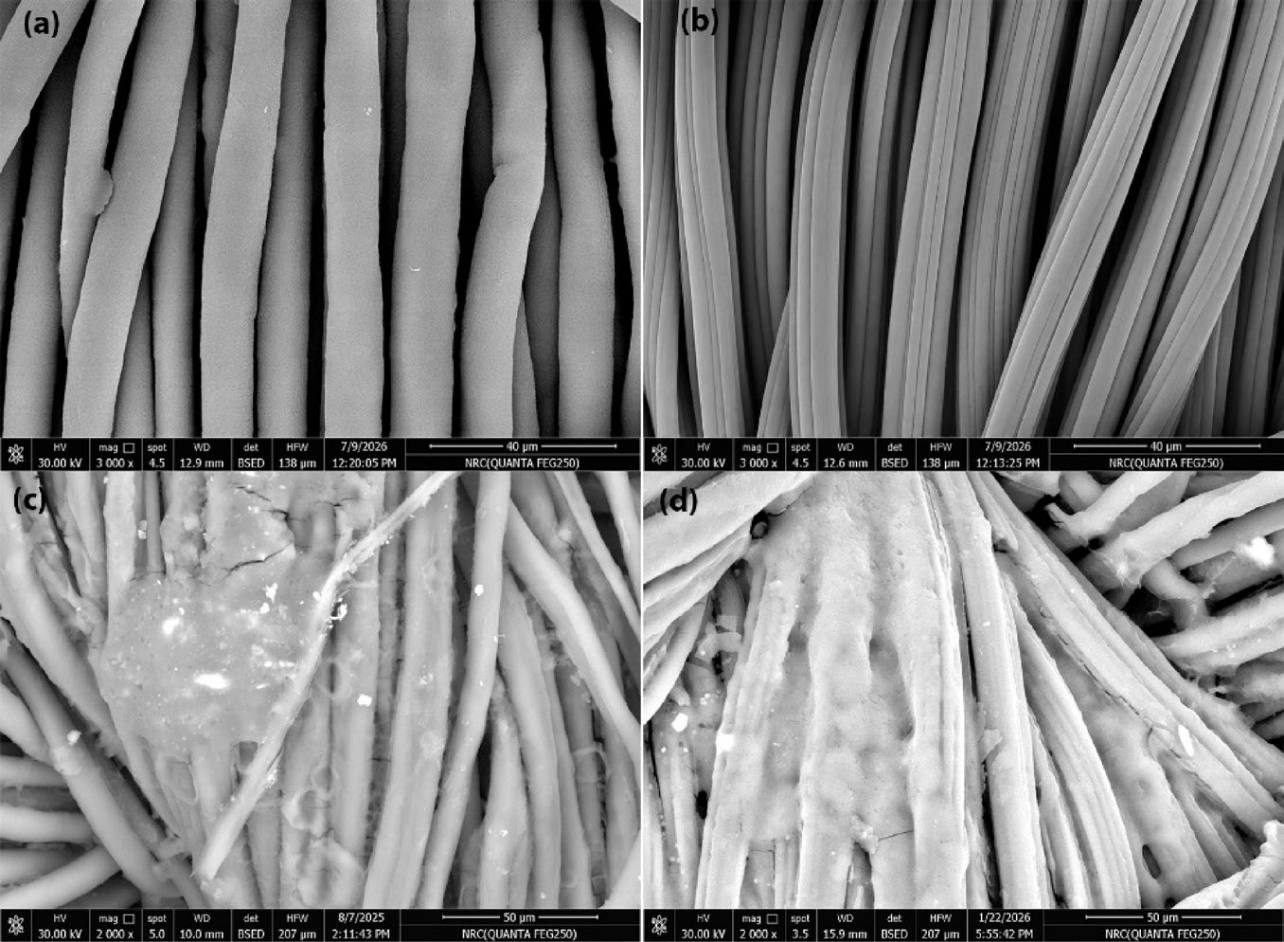
SEM images of: i) untreated Tencel and bamboo fabric samples (**a** & **b**), ii) pre-modified with gelatin/β-cyclodextrin biopolymers → post-dyed with Natural Brown 6/finished with clove oil (**c** & **d**), respectively.

The surface morphology of untreated and treated regenerated cellulosic fabric samples was detected using electron microscopy (SEM). The SEM micrographs of untreated Tencel and bamboo samples are illustrated in Fig. 3a and b, respectively. The SEM image of the untreated Tencel fabric sample (Fig. 3a) showed a clean and smooth surface with rod-shaped fibers, while the SEM of bamboo viscose (Fig. 3b) showed longitudinal grooves with micro-voids running along the fiber length^45,46^.

On the other hand, the SEM images of simultaneously modified Tencel and bamboo samples with gelatin/β-cyclodextrin biopolymers post-dyed with Natural brown 8 and finished with clove oil are presented in Fig. 3c and d, respectively. The images clearly illustrate that the surface of both samples exhibited a continuous coating layer covering the fibers, confirming the deposition of gelatin/β-cyclodextrin biopolymers onto the fabric surface.

### EDX analysis spectra and elemental composition

**Fig. 4:**
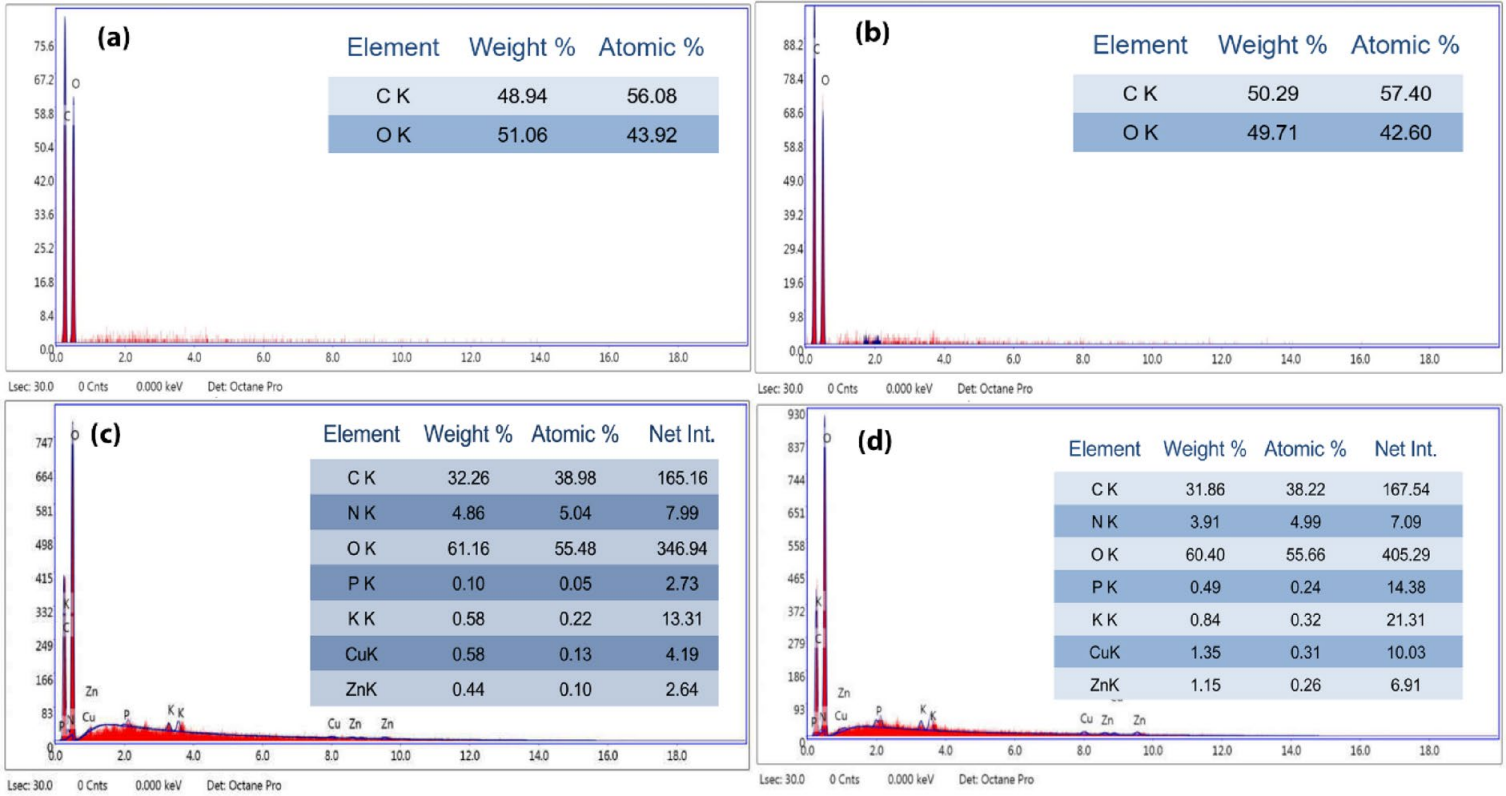
EDX spectra and elemental composition of: i) untreated Tencel and bamboo fabric samples (**a** & **b**), ii) pre-modified with gelatin/β-cyclodextrin biopolymers → post-dyed with Natural Brown 6/finished with clove oil (**c** & **d**) respectively.

The elemental composition and chemical characterization of untreated and treated regenerated cellulosic fabric samples were determined using energy-dispersive X-ray spectroscopy and presented in Fig. 4. The EDX spectra of untreated Tencel and bamboo viscose showed only two characteristic peaks of carbon and oxygen corresponding to the chemical composition of the regenerated cellulosic fabric (Fig. 4a, b). While the patterns of modified Tencel and bamboo samples with gelatin/β-cyclodextrin biopolymers post-dyed with Natural brown 8 and finished with clove oil (Fig. 4c, d) exhibited peaks of carbon (C) and oxygen (O) and additional distinct nitrogen (N) signals, along with trace elemental signals attributed to the constituents of clove essential oil, which were detected in the treated samples. The presence of nitrogen (N), which is absent in untreated regenerated cellulosic fibers, provides clear evidence of successful immobilization of the gelatin/β-cyclodextrin coating on the fabric surface.

Conclusively, SEM images and EDX patterns confirm the successful eco-friendly surface modification that created a functionalized coating onto the regenerated cellulose fibers, which in turn enhanced the post-simultaneous dyeing and functional finishing process using natural dyes and essential oils.

### Surface modification gelatin/β-cyclodextrin post-dyeing and functional finishing

**Fig. 5:**
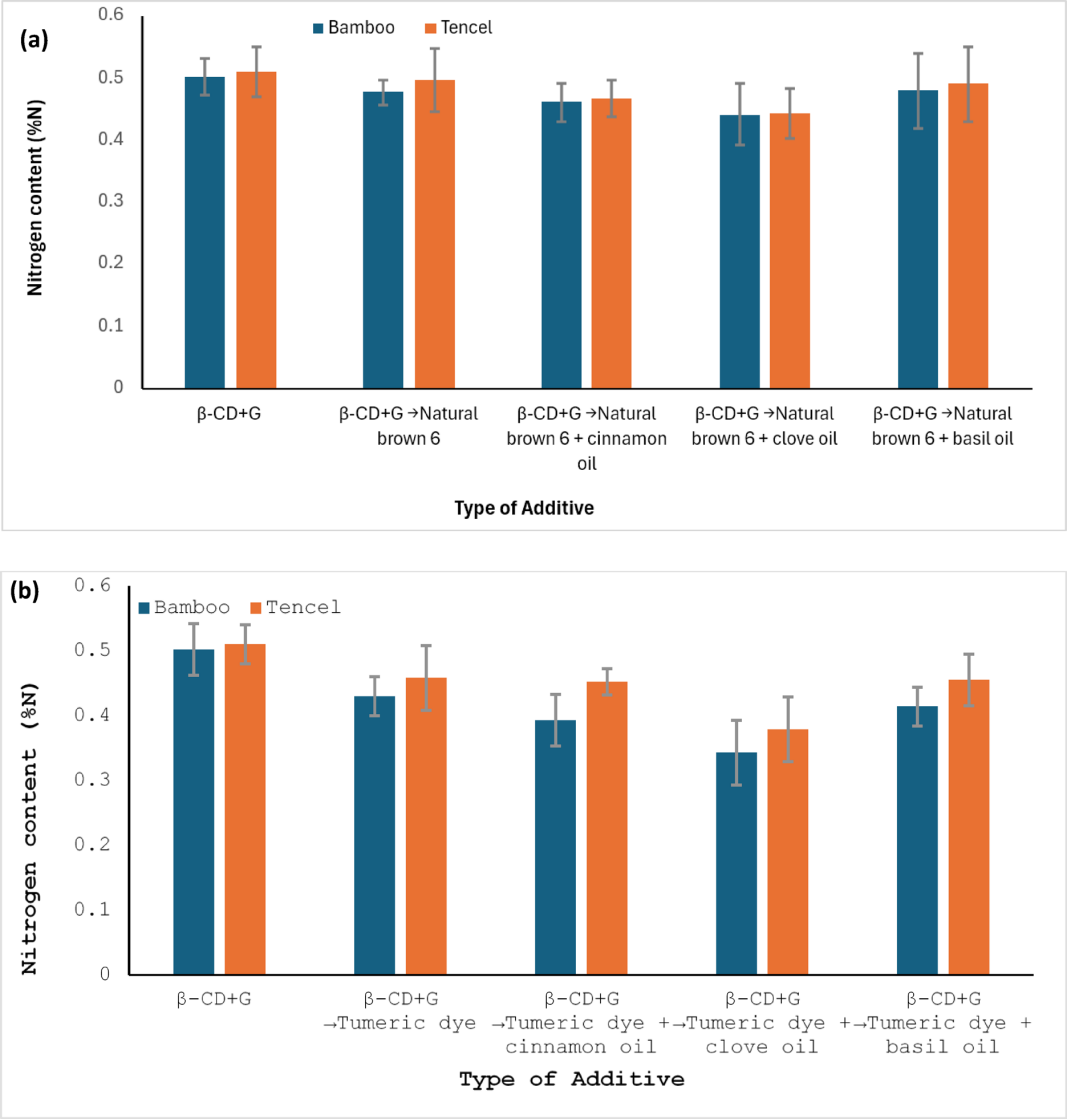
Nitrogen content of regenerated cellulosic fabric samples modified with gelatin/β-cyclodextrin biopolymers (G + β-CD), post-dyed/finished with essential oils (**a**) Natural Brown 6, (**b**) Turmeric dye.

Figure 5 demonstrates that incorporation of gelatin/β-cyclodextrin biopolymers into the finishing formulation using CA (20 g/L), as an ester crosslinking agent, and SHP (10 g/L), as an appropriate ester catalyst for crosslinking of cellulose-OH, results in an increase in %N (from Zero to 0.510 and 0.502 for Tencel and bamboo, respectively), as a direct consequence of loading of the nominated bio-polymers onto the ester-crosslinked cellulose structure during the thermofixation, forming a stable functional layer capable of enhancing post-natural dyeing and finishing with essential oils.

The data presented in Fig. 5 also show that post-dyeing with natural dyes and the inclusion of essential oils into the hydrophobic cavities of β-cyclodextrin grafted onto the pre-modified Tencel and bamboo samples result in a decrease in the nitrogen content (%N) of the dyed/finished fabric samples. The variation in %N is governed by: i) treatment sequence, modified with gelatin/β-cyclodextrin > modified with gelatin/β-cyclodextrin → post-dyeing with natural dyes > modified with gelatin/β-cyclodextrin → combined post-dyeing with natural dyes/finishing with essential oil, ii) type and chemical structure of natural dyes, Natural brown 6 > turmeric dye, iii) essential oil composition, molecular size, and extent of post-inclusion, basil oil > cinnamon oil > clove oil, and iv) type of cellulosic substrates, Tencel > bamboo.

**Scheme 1:**
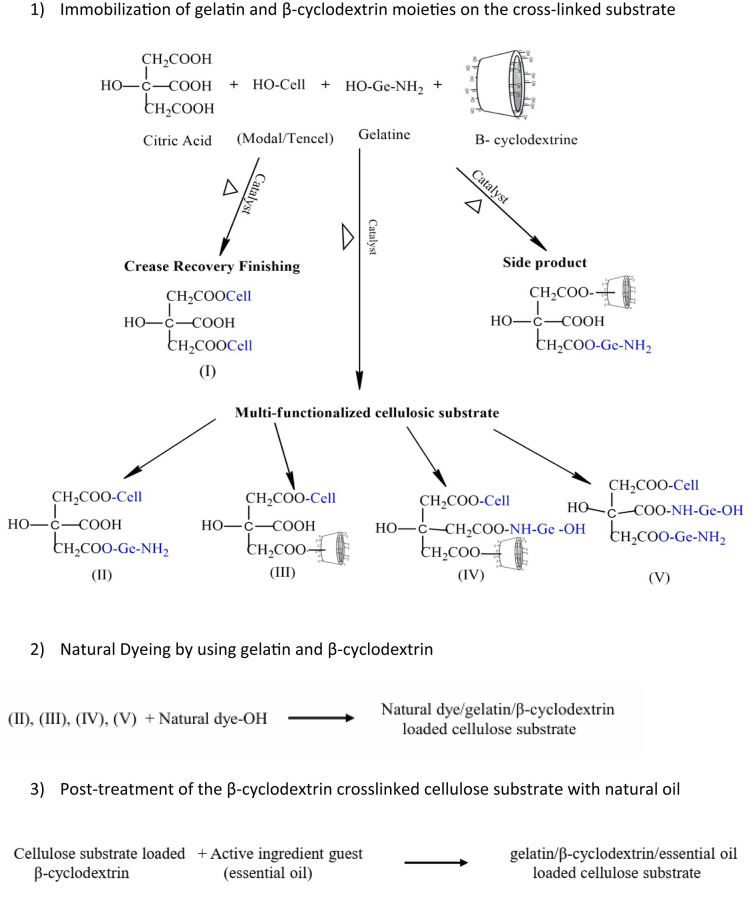
Reaction scheme of premodification of cellulosic fabric with gelatin/β-cyclodextrin biopolymers followed by post-dyeing with natural dyes and functional finishing with essential oils.

The following scheme illustrates the possible interactions among newly introduced active sites onto or within the surface-modified fabrics with gelatin/β-cyclodextrin biopolymers, natural dyes, and essential oils (Scheme 1).

### Natural-dyeing properties

**Fig. 6:**
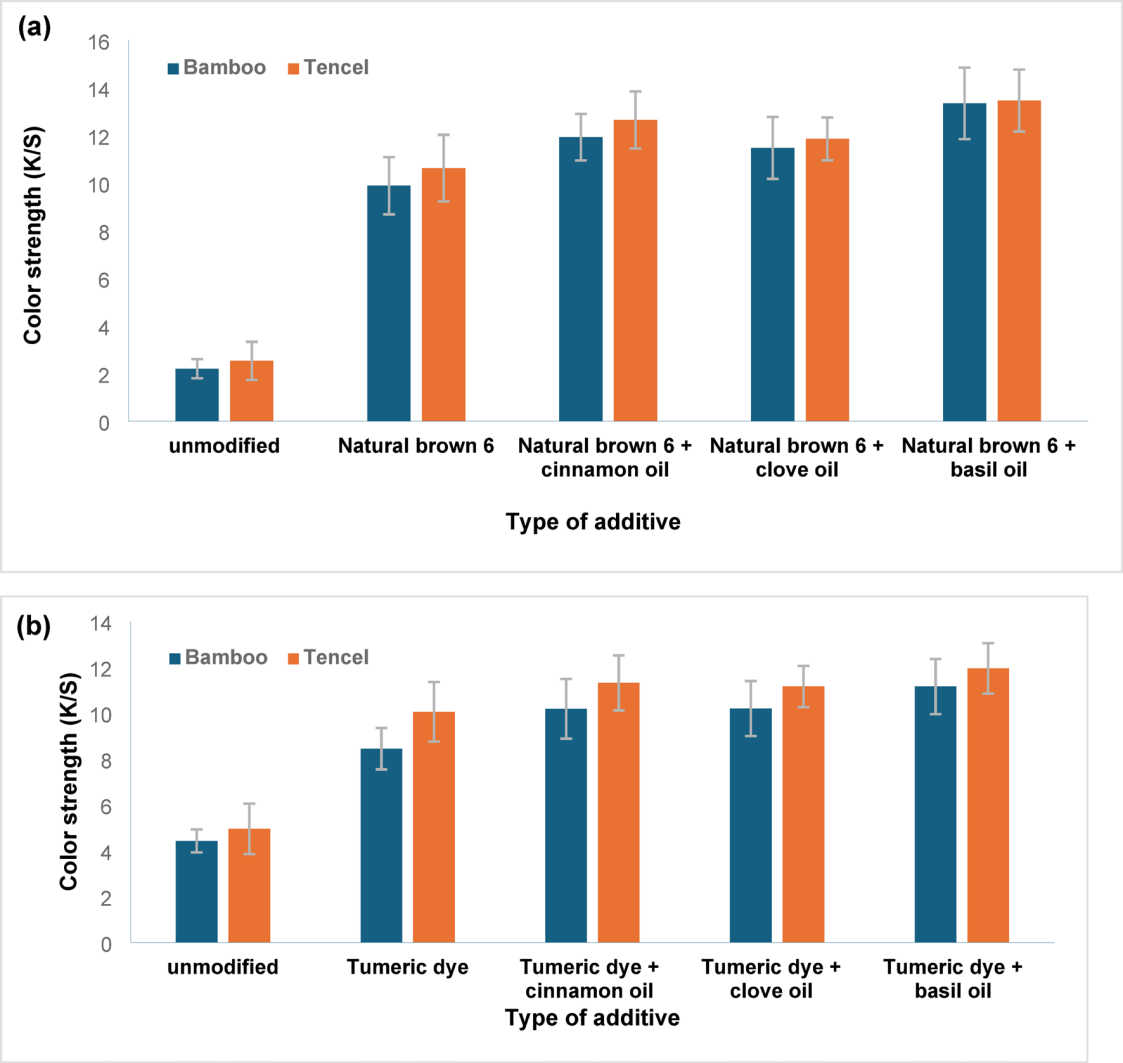
Color strength (K/S) of regenerated cellulosic fabric samples pre-modified with gelatin/β-cyclodextrin biopolymers post-dyed/finished with essential oils (**a**) Natural Brown 6, (**b**) Turmeric dye.

The enhancement in dyeing properties of pre-modified Tencel and bamboo fabric samples with β-cyclodextrin/gelatin biopolymers, then post-simultaneous dyeing and functional finishing via natural dye/essential oil formulation, was expressed by K/S, and colorimetric analysis is presented in Fig. 6 and Table 2

**Table 2:** Effect of combined dyeing and functional finishing on coloration properties of treated regenerated cellulosic fabrics.

Type of dye	Type of additives	Bamboo			Tencel		
		L	a*	b*	L	a*	b*
Natural Brown 6 dye	Dye	54.39	9.50	24.35	53.96	8.37	24.81
	Dye + Cinnamon	49.71	10.18	27.68	50.56	9.54	27.36
	Dye + Clove	44.50	9.83	25.75	49.47	9.02	23.33
	Dye + Basil	45.95	7.24	24.93	49.49	8.46	25.27
Turmeric dye	Dye	77.12	3.82	77.67	77.57	0.38	75.53
	Dye + Cinnamon	75.02	10.1	78.14	77.00	0.52	78.32
	Dye + Clove	74.14	0.97	73.75	75.94	0.63	70.86
	Dye + Basil	75 .66	2.73	77.76	76.69	1.88	78.86

Figure 6 shows that the pristine Tencel and bamboo fabrics exhibited K/S values of 2.53 and 2.20, respectively, when dyed with Natural Brown, and 4.95 and 4.42, respectively, when dyed with turmeric. These results indicate that the untreated regenerated cellulosic fabrics possess low to moderate dye affinity toward the investigated natural dyes, with turmeric exhibiting higher color strength than Natural brown 6 under the same dyeing conditions.

Figure 6 demonstrates that the modified Tencel and bamboo fabrics with gelatin/β-cyclodextrin, in the presence of a citric acid/SHP crosslinking system, significantly enhanced the color strength (K/S) of the regenerated cellulosic fabrics. The K/S values increased to 10.62 and 8.99 for Tencel and bamboo fabrics, respectively, when dyed with Natural Brown 6, while values reached 10.05 and 8.44 for fabrics dyed with turmeric dye.

The enhancement in color strength can be attributed to the synergistic effect of gelatin/β-cyclodextrin premodification, which improved the dyeability of Tencel and bamboo fabrics and the fixation of natural dye and essential oil molecules within the biopolymeric network. The incorporation of gelatin increased the dye affinity by introducing additional active binding sites and enhancing dye adsorption onto the fabric surface^47,48^. Moreover, gelatin forms a thin layer onto the cellulosic fibers, transforming the surface from anionic to cationic, thereby strengthening the electrostatic interactions between the fibers and dye molecules, resulting in improved dye uptake and fixation^48^. Simultaneously, β-cyclodextrin acted as a host molecule capable of forming inclusion (host–guest) complexes with the hydrophobic constituents of the natural dyes and essential oils, providing additional immobilization sites within the crosslinked biopolymers^22^. Furthermore, the incorporation of essential oils may enhance dye–fiber interactions, resulting in deeper and more uniform color shades^22,49,50^.

Moreover, fabric samples dyed with Natural Brown 6 or turmeric dye in the presence of clove, cinnamon, or basil oils exhibited a higher depth of shade compared with samples dyed without oils (Fig. 6). The higher depth of shade in the presence of essential oil is attributed to hydrophobic interactions, which promote the co-encapsulation of both natural dyes and hydrophobic essential oil components within β-cyclodextrin cavities, thereby enhancing the overall affinity of the natural dye/oil formulation for the fabrics^32,33^. The improvement in K/S values followed this descending order: basil oil ≥ cinnamon oil > clove oil > Natural dye alone, regardless of dyestuff and dyed fabric. The variation in the K/S of the dyed fabric samples may be attributed to the chemical compositions of the essential oils, which can act as biomordants, enhancing the extent of natural dye fixation onto/within the pre-modified fabric samples^51^. The main components of basil oil are linalool (43–48%), methyl chavicol (estragole) (13–14%), and 1,8-cineole (6–7%). Clove oil is composed mainly of eugenol, β-caryophyllene, and α-humulene. On the other hand, cinnamon oil is composed of cinnamaldehyde (71.50%)^52,53^.

On the other hand, dyed/finished Tencel fabric samples generally exhibited higher K/S values than bamboo, despite both fabrics exhibiting excellent absorbency and dye uptake. This can be attributed to the nature and structural differences between the two regenerated cellulose substrates, the amorphous-to-crystalline ratio, and accessibility. Tencel consists of nanofibrils and nanochannels, resulting in superior and more even dyeing^54^.

The change in coloration behavior of simultaneous dye/finished regenerated cellulosic fibers after surface modification was evaluated, and the data are given in Table 2. The L* values (lightness) for both Tencel and bamboo samples were significantly reduced after incorporating essential oils into the dyeing/finishing formulation, indicating deeper and darker shades compared to the dyed samples without oils. The degree of reduction in L* value can be attributed to the density and composition of the used oil^50^.

On the other hand, Color fastness is broadly regarded as a critical indicator of product quality from a buyer’s perspective. Table 3 presents the impact of natural oil on the rubbing and washing fastness of functionalized and post-dyed fabrics, treated with a β-cyclodextrin/gelatin system in the presence of turmeric and natural brown dyes. Within textile testing laboratories, color fastness is systematically evaluated by exposing samples to standard conditions and visually comparing color change or staining against the grey scale. However, this method is subjective and may lead to inconsistent results. In this study, an automated grading approach based on CIELab color difference values is proposed to objectively assess color change and staining, using ISO 105-A02 and ISO 105-A03 as reference scales.

**Table 3:** Colour fastness to crocking.

Fabric	Natural Oil	Turmeric dye				Natural brown dye			
		Dry		Wet		Dry		Wet	
		CIELAB difference	Fastness grade	CIELAB difference	Fastness grade	CIELAB difference	Fastness grade	CIELAB difference	Fastness grade
Tencel	Dye	5.92	3/4	8.14	3	4.30	4	6.21	3/4
	Clove	5.73	3/4	7.23	3	4.13	4	5.87	3/4
	Cinnamon	4.13	4	5.91	3/4	3.72	4	4.82	3/4
	Basil	3.15	4	5.32	3/4	2.18	4/5	3.26	4
Bamboo	Dye	6.25	3/4	9.54	3	4.37	4	8.34	3
	Clove	5.81	3/4	8.12	3	4.23	4	7.36	3
	Cinnamon	5.62	3/4	7.31	3	3.86	4	5.37	3
	Basil	4.12	4	6.34	3	3.27	4	4.78	3

From Tables 3 and 4, the results indicate that natural oil treatments positively affect both rubbing and washing color fastness, with performance dependent on fabric type and dye. In the case of color fastness to rubbing, Tencel fabrics showed noticeable improvement, particularly with basil and cinnamon oils, achieving good to very good grades in dry conditions with color differences of rubbed-stained dry cotton cloths (3.15 and 4.13), respectively. Furthermore, wet rubbing remained moderate with color differences (5.32 and 5.91), respectively, of rubbed-stained wet cotton cloths. Bamboo fabrics, however, exhibited limited enhancement rather than Tencel fabrics. The lower rubbing fastness observed for turmeric-dyed fabric may be attributed to the less interaction of curcumin with the fiber surface, and also the high brightness and chromaticity of the yellow color. Even a small amount of transferred curcumin is more visually noticeable on the white rubbing cloth than the transferred brown dye, resulting in a lower apparent rubbing fastness rating. For washing fastness, natural oils significantly improved color change and staining resistance for both fabrics (Cotton, and wool). Tencel samples treated with basil and cinnamon oils exhibited excellent resistance to staining on cotton and wool. Across all treatments, the natural brown dye consistently demonstrated better overall fastness performance than turmeric.

**Table 4:** Colour fastness to washing.

Fabric	Natural dye	Natural oil	Color change		Staining to cotton		Staining to wool	
			CIELAB difference	fastness grade	CIELAB difference	Fastness grade	CIELAB difference	Fastness grade
Tencel	Turmeric dye	Dye	4.83	2/3	5.37	4	5.13	4
		Clove	3.21	3	4.01	4	3.97	4
		Cinnamon	2.04	3/4	3.14	4/5	2.87	4/5
		Basil	1.57	4	2.17	4/5	2.09	4/5
Bamboo		Dye	5.28	2/3	5.28	4	4.97	4
		Clove	3.89	3	4.35	4	3.32	4
		Cinnamon	3.52	3	3.27	4/5	2.25	4/5
		Basil	2.86	3/4	1.96	4/5	1.81	4/5
Tencel	Natural brown 6	Dye	2.45	3/4	4.09	4/5	3.98	4/5
		Clove	1.94	4	3.98	4/5	3.65	4/5
		Cinammon	1.43	4	3.76	4/5	3.26	4/5
		Basil	1.07	4	3.45	4/5	2.36	4/5
Bamboo		Dye	2.32	3/4	4.32	4	4.67	4
		Clove	1.87	4	4.12	4/5	4.21	4/5
		Cinammon	1.34	4	3.84	4/5	3.65	4/5
		Basil	1.12	4	3.65	4/5	3.28	4/5

Finally, the functionalization of the fabric with β-cyclodextrin/gelatin enhances dye uptake through hydrogen bonding and/or π–π stacking interactions^47,55^. In addition, natural oils can be accommodated within the β-cyclodextrin cavity via inclusion complex formation^56,57^. These oils further assist in dye fixation by acting in a mordant-like manner, promoting the formation of more stable dye–fiber complexes^51^. Consequently, this synergistic system strengthens dye binding and leads to improved color fastness of the treated textiles.

### Antibacterial activity of dye/functional finished cellulosic fabrics

**Fig. 7:**
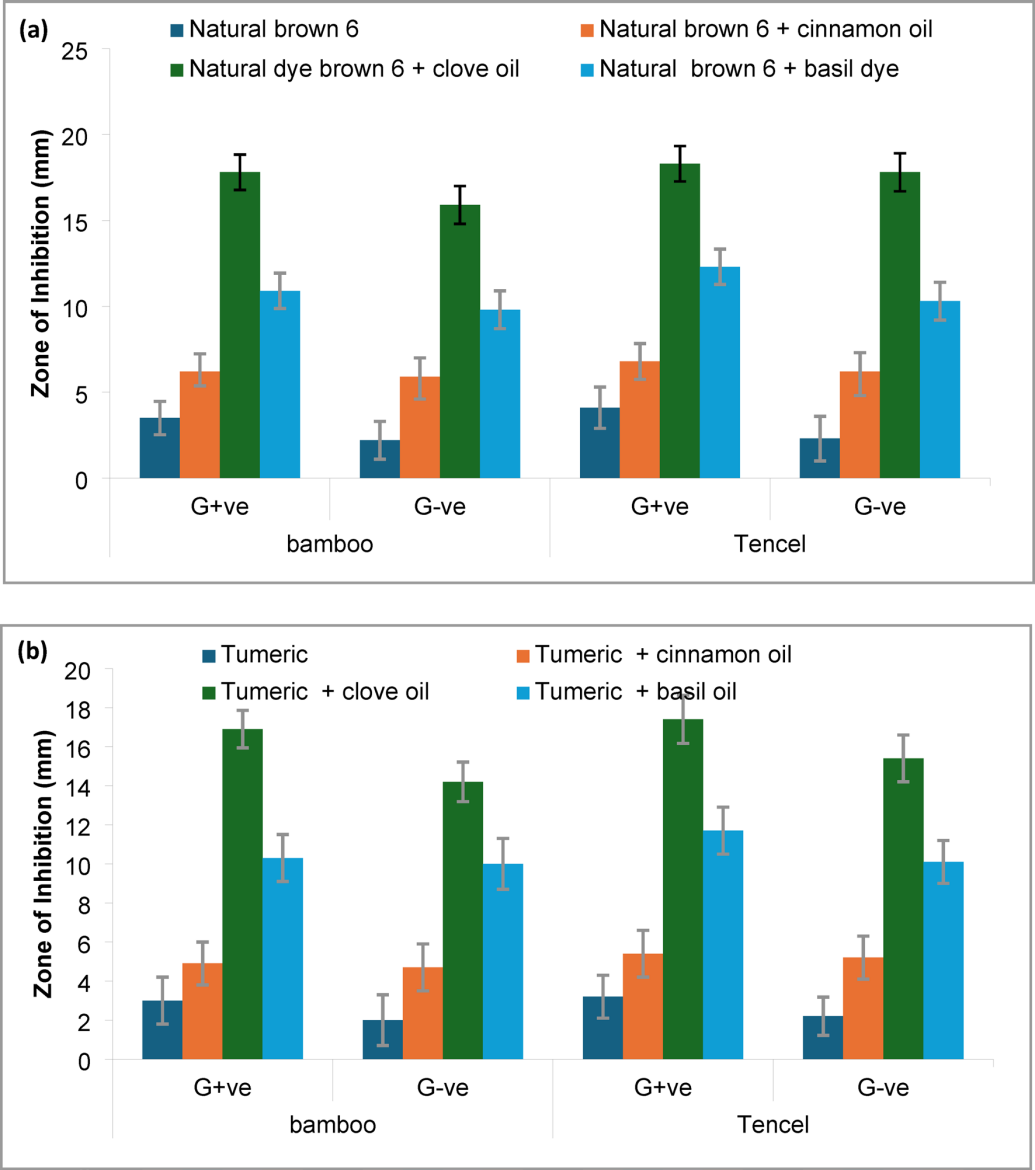
Effect of simultaneous post-natural dyeing /functional finishing, (**a**) Natural Brown 6, (**b**) Turmeric dye, on antibacterial activity (expressed as zone of inhibition) of treated regenerated cellulosic fabrics.

**Fig. 8:**
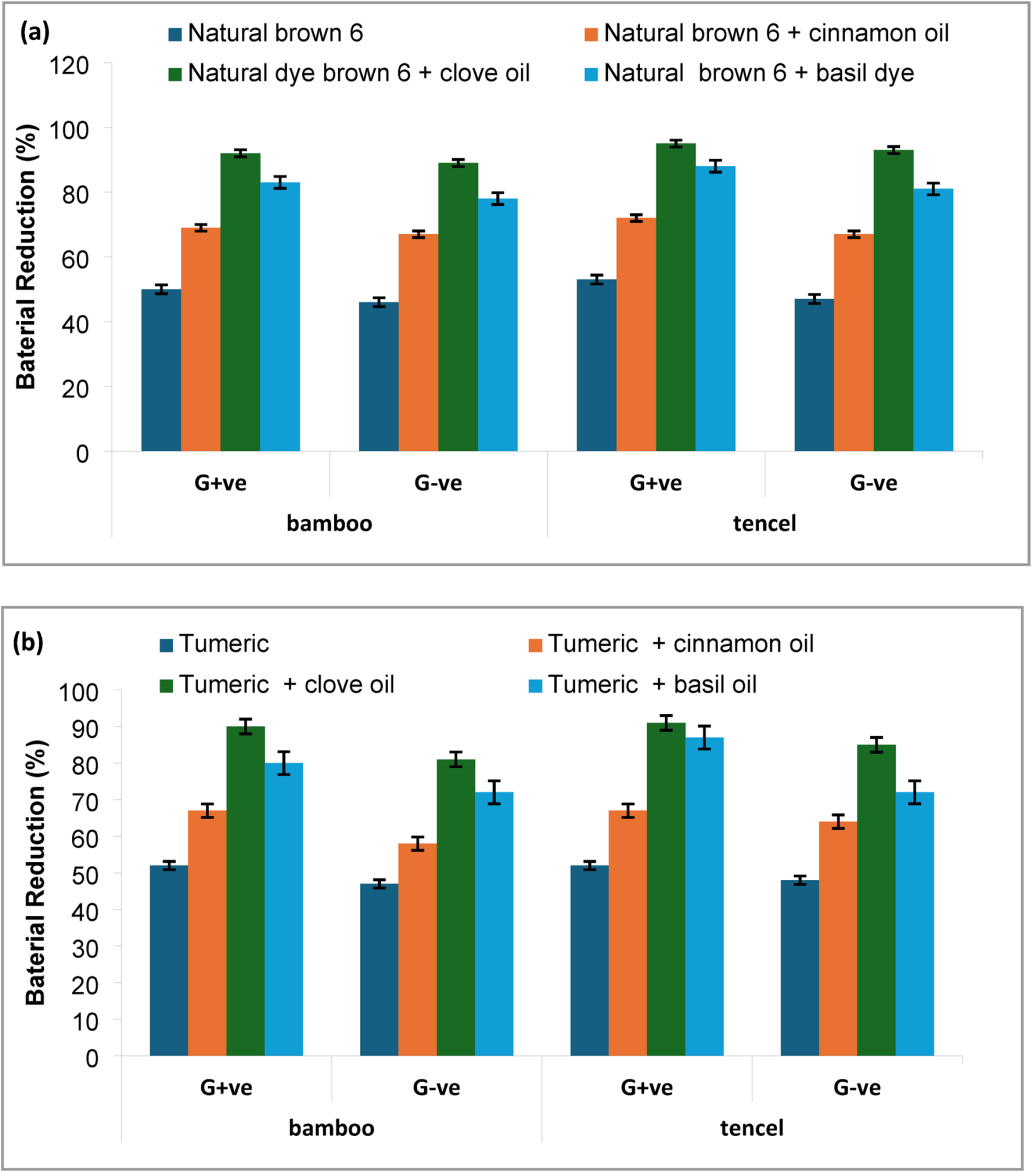
Effect of simultaneous post-natural dyeing /functional finishing, (**a**) Natural Brown 6, (**b**) Turmeric dye, on antibacterial activity (expressed as % bacterial reduction) of treated regenerated cellulosic fabrics.

Figures 7a&b and 8a&b present antibacterial activity of the dyed/finished fabric samples assessed by qualitative (zone of inhibition) and quantitative (percent reduction) test methods. Untreated Tencel and bamboo fabrics showed no antibacterial activity against the tested Gram-positive *Staphylococcus aureus* and Gram-negative *Escherichia coli* bacteria*.*

The data in Figs. 7 and 8 revealed that the fabric samples modified with gelatin/β-cyclodextrin and post-dyed with natural dyes exhibited noticeable improvement in antibacterial performance regardless of the type of natural dye or the type of regenerated cellulosic fabric. The improvement in antibacterial activity of the dyed fabric samples with turmeric and Natural Brown 6 dye can be attributed to the intrinsic bioactive components, namely phenolic, flavonoid, and tannin compounds, found in their compositions. Regarding the type of natural dye used, Natural Brown 6-dyed fabric samples showed higher antibacterial activity than turmeric-dyed samples in qualitative and quantitative evaluation. The differences in antibacterial activity of the natural dyes are due to their chemical compositions, which in turn affect their modes of action in disrupting microbial cell membranes and inhibiting bacterial growth, as well as their extent of release^58,59^. The main compound in Natural brown 6 is based on chebulinic acid. Chebulinic acid is a hydrolysable tannin characterized by remarkable antibacterial activity capable of inhibiting bacterial growth, causing deformation and cell shrinkage, damaging the structural integrity by increasing the release of nucleic acids and proteins, which are the main components of intercellular^60^. Turmeric is rich in phytochemical components with broad biological activities. The major bioactive compound is curcumin, able to suppress bacterial DNA replication and virulence factors while damaging bacterial cell walls and membranes, allowing cellular contents to leak out^61^.

The incorporation of essential oils (20 g/L) during the combined dyeing/finishing stage resulted in a remarkable improvement in antibacterial activity of the finished samples, regardless of the type of fabric samples. Cellulosic samples treated with clove oil exhibited the highest antibacterial activity expressed as a zone of inhibition (Fig. 7) and antibacterial reduction (95%) for both tested bacteria (Fig. 8). Samples treated with cinnamon and basil oils exhibited excellent antibacterial activity, but lower than that of clove oil. The differences in their antibacterial behavior are mainly due to the major content of bioactive compounds in their composition, as eugenol is the main component in clove oil, cinnamaldehyde is the main component in cinnamon oil, and linalool is the main component in basil oil^62^.

The enhancement in antibacterial performance can be attributed to the synergistic effect of the gelatin/β-cyclodextrin modification system. Gelatin functions as a bio-mordant, increasing the affinity of regenerated cellulosic fabrics toward natural dyes through the introduction of additional binding sites and improved dye fixation^38^. Simultaneously, β-cyclodextrin acts as a host molecule by forming inclusion complexes with natural dye molecules and hydrophobic constituents of the essential oil. This host–guest interaction enhances the loading and retention of the bioactive compounds within the β-cyclodextrin cavities and enables their controlled release from the fabric surface, thereby providing prolonged and more effective antibacterial activity^33,50^.

Furthermore, Tencel fabric samples exhibited better antibacterial activity than bamboo viscose samples. The variation in their antibacterial activity is due to their origin, cellulose composition, the extent of distribution of gelatin/β-cyclodextrin layer onto/within the fiber surface, the fixation of gelatin/β-cyclodextrin, and the extent of accessible cavities for encapsulating oils^46^.

The results presented in Figs. 7 and 8 also demonstrate that the antibacterial efficacy of the treated fabrics depended on the type of pathogenic bacteria. *Staphylococcus aureus* (G + ve) bacteria exhibited higher susceptibility to the treated samples than *Escherichia coli* (G-ve) bacteria. This difference can be attributed to the distinct structural characteristics of their cell wall membrane. Gram-positive bacteria possess a relatively porous peptidoglycan layer that facilitates the penetration of antimicrobial agents, while Gram-negative bacteria are protected by an additional outer membrane enriched with lipopolysaccharides, which acts as an effective permeability barrier and limits the diffusion of bioactive compounds into the bacterial cells^63^.

### UV-protection properties

**Fig. 9:**
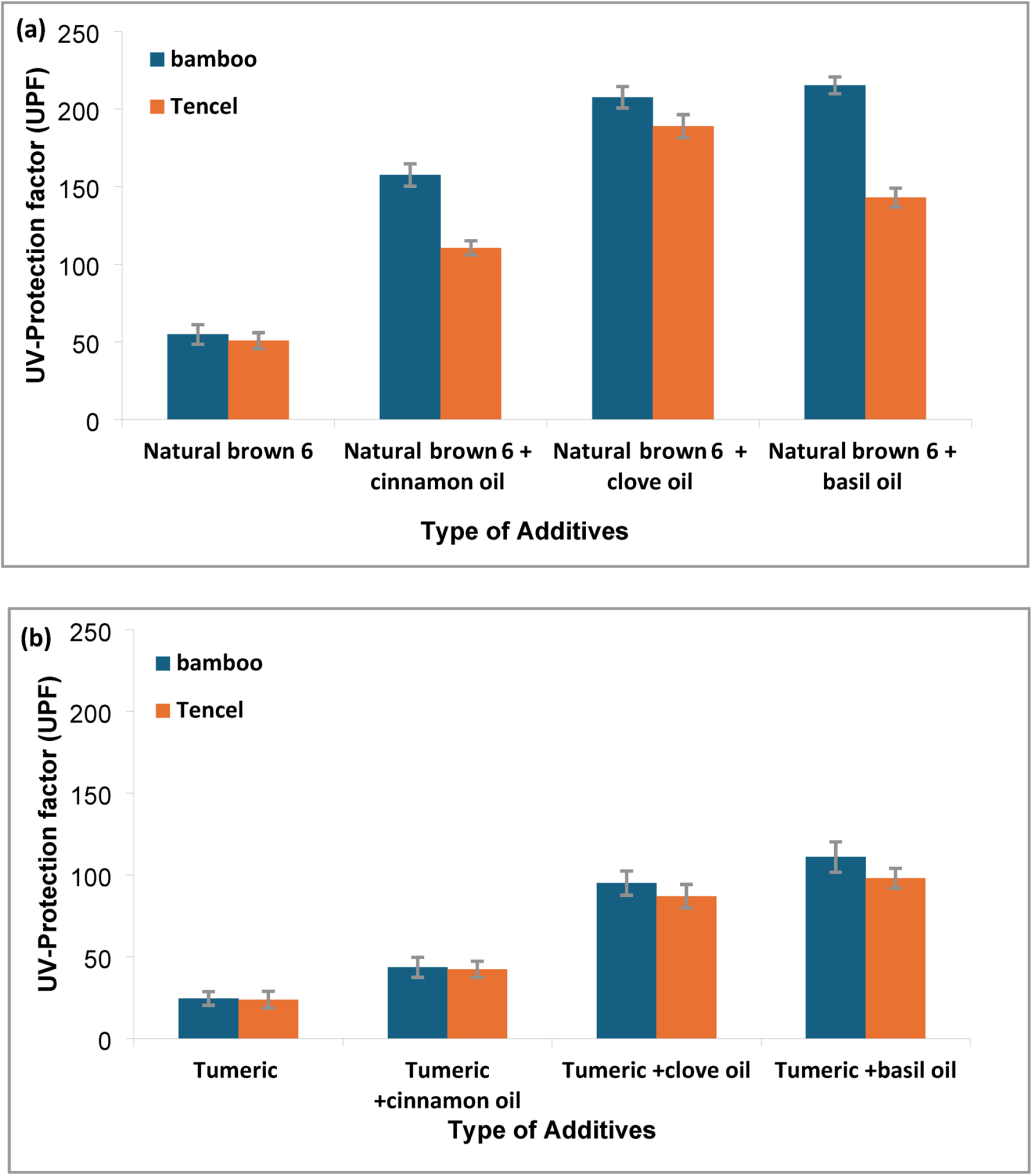
Effect of simultaneous post-natural dyeing /functional finishing, (**a**) Natural Brown 6, (**b**) Turmeric dye, on UV-protection properties of treated regenerated cellulosic fabrics.

Ultraviolet protection factor (UPF) measurements of modified regenerated cellulosic fabrics post-dyed with natural dyes in the absence and presence of essential oils are given in Fig. 9 (a&b). The untreated Tencel and bamboo viscose exhibit low UPF values due to the cellulosic nature, light weight, and fabric structure.

The gelatin/β-cyclodextrin-modified regenerated cellulosic fabrics post-dyed with the natural dyes Natural Brown 6 and turmeric exhibited a marked enhancement in UV- protection factor (UPF) values compared with the untreated ones. The modified bamboo fabrics attained UPF values of 54 and 24 after dyeing with Natural Brown 6 and turmeric, respectively, while Tencel fabrics exhibited UPF values of 50 and 23. The enhanced UV-shielding performance can be attributed to the synergistic effect of gelatin/β-cyclodextrin surface modification and natural dye deposition.

The improvement in the UV-protection of dyed cellulosic fabric samples can be due to coating with a gelatin/β-cyclodextrin layer onto the fiber surface, which in turn reduces the transmission of ultraviolet radiation through the fabric. Furthermore, the natural dyes contribute to UV protection through their intrinsic UV-absorbing properties owing to conjugated aromatic structures and phenolic functional groups, which effectively absorb incident UV radiation. These combined effects significantly improve the UV-protective performance of the treated fabrics, irrespective of the regenerated cellulosic substrate. Additionally, Natural brown 6 -dyed fabric samples showed higher UPF values compared with samples dyed with turmeric due to higher color strength (K/S), greater fabric uptake, aromatic chromophores, phenolic hydroxyl groups, and conjugated π-electron systems^64,65^.

Furthermore, incorporation of essential oils into the dyeing/finishing formulation resulted in a remarkable increase in UPF values in dyed/finished fabric samples, regardless of the type of essential oil used or fabric type. The UPF values of all treated samples exceeded 50 (excellent UV-protection). This increase may be due to extra scattering and absorption of UV rays by essential oil components. Regarding the essential oils, basil and clove oils showed better UV-shielding performance compared with cinnamon oil in finished fabrics. The variation in UPF values regarding the type of essential oil used can be attributed to the variation in the composition of the used essential oil and the extent of fixation of the bioactive agents within β-cyclodextrin cavities, as well as their ability to absorb and/or scatter the harmful UV rays^66^.

On the other hand, the results revealed that dyed/finished bamboo fabrics showed slightly higher UV-protection activity than Tencel fabric samples, regardless of treatment formulations. This increment is due to the variation in fabric nature, porosity, crystalline and amorphous regions, as well as the extent of modification^46^.

### Physical and comfort properties

Table 5 presents the influence of gelatin/β-cyclodextrin surface modification, followed by concurrent natural dyeing and essential-oil finishing, on the physical and comfort-related performance of regenerated cellulosic fabrics expressed by crease recovery angle (CRA), surface roughness (Ra), and air permeability (AP).

**Table 5:** Effect of combined dyeing and functional finishing on physical and comfort properties of treated regenerated cellulosic fabrics.

Type of dye	Type of additives	Bamboo				Tencel			
		CRA (W + F)^o^	Ra (μm)	AP (cm^3^/cm^2^/sec)	T.S (warp direction) (N)	CRA (W + F)^o^	Ra (μm)	AP (cm^3^/cm^2^/sec)	T.S (warp direction) (N)
Without	untreated	162 ± 1.50	11.09 ± 0.05	35.60 ± 0.71	443.65 ± 0.45	170 ± 1.3	10.54 ± 0.07	45.2 ± 0.68	396.9 ± 0.63
	Blank	210 ± 1.00	15.80 ± 0.15	28.70 ± 0.86	451.98 ± 1.1	215 ± 1.0	14.76 ± 0.09	35.30 ± 88	443.95 ± 1.0
Natural Brown 6	Dye	218 ± 1.00	17.96 ± 0.26	27.90 ± 0.97	482.97 ± 0.73	225 ± 1.0	16.03 ± 0.14	31.0 ± 0.79	458.65 ± 0.8
	Dye + Cinnamon	232.5 ± 1.5	13.14 ± 0.17	25.30 ± 1.0	457.63 ± 0.47	238 ± 1.3	12.55 ± 0.11	25.23 ± 0.75	440.54 ± 0.65
	Dye + Clove	226.5 ± 1.5	13.15 ± 0.21	24.00 ± 0.95	450.32 ± 0.69	229 ± 1.3	12.47 ± 0.13	28.8 ± 0.84	443.32 ± 1.1
	Dye + Basil	239 ± 0.76	12.70 ± 0.11	25.00 ± 0.84	468.40 ± 0.64	246 ± 1.0	11.40 ± 0.16	29.0 ± 0.87	445.53 ± 1.3
Curcumin	Dye	210 ± 1.00	15.76 ± 0.13	22.30 ± 0.78	510.14 ± 0.92	215 ± 0.95	14.37 ± 0.21	24.6 ± 0.62	459.44 ± 0.85
	Dye + Cinnamon	244.5 ± 1.5	12.63 ± 0.27	20.02 ± 0.70	440.32 ± 0.58	241 ± 1.5	11.55 ± 0.30	23.4 ± 0.70	385.58 ± 0.56
	Dye + Clove	230 ± 1.30	12.92 ± 0.38	20.50 ± 0.82	458.40 ± 0.89	232 ± 0.85	11. 98 ± 17	21.20 ± 0.74	409.60 ± 1.0
	Dye + Basil	251 ± 0.87	11.66 ± 0.30	20.40 ± 0.71	490.60 ± 0.85	257 ± 0.80	11.08 ± 0.21	21.8 ± 0.76	410.56 ± 1.0

Untreated bamboo and Tencel fabrics exhibited relatively low CRA values (162° and 170°, respectively), indicating limited resistance to wrinkling. The increment in the CRA values is attributed to the treatment sequences and follows the decreasing order: gelatin/β-cyclodextrin premodification → natural dyeing/essential-oil finishing > gelatin/β-cyclodextrin premodification → natural dyeing > gelatin/β-cyclodextrin premodification > None. Improvement in crease recovery can be attributed to the presence of citric acid as a crosslinking agent, along with sodium hypophosphite as a catalyst, resulting in the formation of ester crosslinks between carboxyl groups of citric acid and hydroxyl groups in cellulose, enhancing fabric resiliency and structure integrity^67^. Moreover, inclusion of gelatin in the finishing bath formulation can improve the flexibility and reduce the rigidity that might occur from the citric acid crosslinking agent^48^.

Incorporation of essential oils (20 g/L) into dyeing/finishing bath resulted in further improvement in the crease recovery of dyed/finished regenerated cellulosic fabric samples, regardless of the type of cellulosic fabric. Essential oils encapsulated within β-cyclodextrin cavities create additional interactions that enhance flexibility by reducing friction during bending and folding. The crease recovery angle reached 251 and 257 for bamboo and Tencel fabric samples when basil oil was used. On the other hand, Tencel samples generally showed slightly higher CRA values than bamboo, likely due to their higher fibre uniformity and structural integrity^46^.

Furthermore, both bamboo and Tencel fabrics are characterized by a soft-touch feel; however, Tencel fabrics are smoother with a silky texture (10.54 μm), compared with bamboo (11.09 μm). The variation in their smooth/soft touch character is attributed to their fiber structure and production process. The surface roughness increased after gelatin/β-cyclodextrin premodification and dyeing with natural dyes. This increase is related to the deposition of the coating layer and dye molecules onto the fabric surfaces. Then, the incorporation of essential oils resulted in a significant reduction in roughness value (Ra) and an enhancement in surface smoothness, reaching 11.08 μm and 11.66 μm for Tencel and bamboo, respectively. The enhancement in the smoothness of dyed/finished fabric samples may be due to film formation and the lubricant effect created by essential oil components within the biopolymers^48^.

On the other hand, air permeability decreased after chemical modification and dyeing/finishing formulation compared with untreated fabrics. This reduction is expected due to partial filling of inter-fiber pores by the gelatin/β-cyclodextrin coating and dye/essential oil deposits, which slightly obstruct airflow pathways. The decline is more pronounced in curcumin-treated samples. However, this reduction in air permeability remains within an acceptable range without compromising the comfort and breathability of the dyed/finished samples. Moreover, bamboo samples showed slightly higher AP values, regardless of the types of dyes and finishing formulation, due to the fabric structure and porosity^67^.

Additionally, tensile strength of treated fabric samples is governed by the treatment sequence. The decrease in tensile strength showed the following descending order: gelatin/β-cyclodextrin → post-dyed with natural dyes > gelatin/β-cyclodextrin → post-dyed with natural dyes /finished with essential oil > gelatin/β-cyclodextrin > untreated, regardless of the type of dye or essential oil. The incorporation of gelatin and β-cyclodextrin within the modification formulation, along with citric acid/SHP, as a crosslinking system, results in slight enhancement in the mechanical properties. Further increase in tensile strength was observed after post-dyeing with natural dyes, especially turmeric dye. The improvement may be attributed to the formation of additional intermolecular interactions and chemical linkages among gelatin/β-cyclodextrin/dyes molecules and the cellulose chain, thus enhancing the fiber cohesion, which results in keeping mechanical properties intact^68^. However, incorporation of essential oil in dyeing/finishing formulation results in a trivial decrease in the tensile strength of the obtained dyed/finished fabric samples.

### Durability to wash

Table 6 records the changes in antimicrobial activity, expressed as zones of inhibition (mm), UV-protection functionality, expressed as UPF values, and color strength, expressed as K/S values, of selected samples after 10 successive washing cycles. The data demonstrate that the samples retained noticeable antibacterial and UV-protective properties even after 10 washing cycles, accompanied by a slight reduction in color strength. The durability of the imparted functional properties can be attributed to sustained retention and controlled release of the encapsulated bioactive agents, their inherent chemical composition and stability, and their interaction with the modified fabric matrix.

**Table 6:** Durability of imparted functional properties and color strength to wash.

Type of fabric	Type of dye	Type of additives	Antibacterial activity (ZI, mm)				UPF		K/S	
			*S. aureus* (G + ve)		*E. coli* (G-ve)		0	10 wash	0	10 wash
			0	10 wash	0	10 wash				
Bamboo	Natural Brown 6	Dye	3.5	2.1	2.2	1.9	54.5	35.9	9.88	7.11
		Dye + Cinnamon	6.2	4.8	5.9	4.5	157.5	77.3	11.92	9.60
		Dye + Clove	17.8	13.3	15.9	13.0	207.6	98.0	11.47	8.40
		Dye + Basil	10.9	9.2	9.8	8.4	215.5	144.3	13.34	11.24
	Turmeric	Dye	3	1.9	2	1.7	24.5	19.1	8.44	5.77
		Dye + Cinnamon	4.9	3.8	4.7	3.2	43.5	30.6	10.18	7.19
		Dye + Clove	16.9	12.9	14.2	12.5	95	54.2	1019	4.38
		Dye + Basil	10.3	9.1	10.0	8.2	111	74.8	11.15	7.88
Tencel	Natural Brown 6	Dye	4.1	2.3	2.3	2.1	50.8	32.6	10.62	7.88
		Dye + Cinnamon	6.8	5.2	6.2	4.8	110.6	64.8	12.64	9.76
		Dye + Clove	18.3	13.9	18.8	12.7	189	80.5	11.85	9.16
		Dye + Basil	12.3	10.1	10.3	9.5	143	106	13.46	12.17
	Turmeric	Dye	3.2	2.1	2.2	1.8	23.5	13.6	10.05	6.11
		Dye + Cinnamon	5.4	4.0	5.2	3.6	42.5	27.3	11.31	7.38
		Dye + Clove	17.4	13.1	15.4	12.9	87	45.6	11.15	4.39
		Dye + Basil	11.7	9.3	10.1	8.4	98	49.3	11.94	8.12

## Conclusion

This study aimed to develop sustainable multifunctional regenerated cellulosic fabrics through eco-friendly surface modification using gelatin and β-cyclodextrin biopolymers, followed by concurrent natural dyeing and essential-oil functional finishing. FT-IR spectra confirmed the chemical modification of the treated fabric through the appearance and quantitative increase in the characteristic functional groups of gelatin/β-cyclodextrin and natural dyes, as evident by the percentage increase in peak height compared to untreated fabric. Surface morphology and elemental composition were also analyzed using scanning electron microscopy (SEM) and energy-dispersive X-ray spectroscopy (EDX), confirming the formation of uniform biopolymer coatings and the successful incorporation of the functional finishing agent.

The pre-modification process showed enhancement in coloration properties expressed by K/S for post-dyed/finished cellulosic fabrics. Moreover, the post-dyed/finished fabric samples exhibited significant improvement in antibacterial activity against both Gram-positive (*Staphylococcus aureus*) and Gram-negative (*Escherichia coli*) bacteria, attributed to the synergistic effect of the natural dye and essential oils. Fabric samples dyed with Natural brown 6 exhibited higher antibacterial activity than samples dyed with turmeric dye. The effect of essential oils on the imparted antibacterial activity showed the following descending order: Clove > basil > cinnamon > None. Regarding the cellulosic type, Tencel fabric samples exhibited better antibacterial activity than bamboo fabric samples.

Additionally, post-dyed/finished fabric samples showed excellent UV protection performance, especially when essential oils were incorporated in the finishing bath formulation due to the synergistic effect of natural dyes and essential oils in absorbing and scattering harmful UV rays. On the other hand, physical properties expressed in CRA and surface roughness of the dye/finished fabric improved while keeping tensile strength and air-permeability properties at an acceptable level.

Overall, the integration of gelatin/β-cyclodextrin biopolymers surface modification, followed by simultaneous natural dyeing and essential oil finishing, proved to be an efficient green strategy for producing durable, sustainable added-value products characterised by combined comfort and protective properties along with aesthetic appeal. This approach presents a promising route for developing eco-friendly multifunctional regenerated cellulosic fabrics.

## Data Availability

All data generated or analyzed during this study are included in this article.
